# Survey of activity pacing across healthcare professionals informs a new activity pacing framework for chronic pain/fatigue

**DOI:** 10.1002/msc.1421

**Published:** 2019-08-20

**Authors:** Deborah Antcliff, Anne‐Maree Keenan, Philip Keeley, Steve Woby, Linda McGowan

**Affiliations:** ^1^ Physiotherapy Department, Bury and Rochdale Care Organisation Northern Care Alliance NHS Group Bury UK; ^2^ School of Healthcare University of Leeds Leeds UK; ^3^ School of Human and Health Sciences University of Huddersfield Huddersfield UK; ^4^ Research and Innovation Department Northern Care Alliance NHS Group Salford UK; ^5^ School of Health and Society University of Salford Salford UK; ^6^ Faculty of Science and Engineering Manchester Metropolitan University Manchester UK

**Keywords:** activity pacing, chronic fatigue, chronic pain, survey

## Abstract

**Introduction:**

Activity pacing is considered a key component of rehabilitation programmes for chronic pain/fatigue. However, there are no widely used guidelines to standardize how pacing is delivered. This study aimed to undertake the first stage in developing a comprehensive evidence‐based activity pacing framework.

**Methods:**

An online survey across pain/fatigue services in English National Health Service trusts explored healthcare professionals' opinions on the types/uses of pacing, aims, facets and perceived effects. Data were analysed using descriptive statistics for closed‐ended questions and thematic analysis for open‐ended questions. Purposeful recruitment with a snowball effect engaged 92 healthcare professionals (physiotherapists, occupational therapists, nurses, doctors and psychologists) to the study.

**Results:**

Pacing was highly utilized, with perceived long‐term benefits for patients (*n* = 83, 90.2% healthcare professionals instructed pacing). The most endorsed aim of pacing was “achievement of meaningful activities” (24.5% of ranked votes). The least endorsed aim was “to conserve energy” (0.1% of ranked votes). The most frequently supported facet of pacing was “breaking down tasks” (*n* = 91, 98.9%). The least supported facet was “stopping activities when symptoms increase” (*n* = 6, 6.5%). Thematic analysis showed recurring themes that pacing involved flexibility and sense of choice.

**Conclusions:**

Pacing is a multidimensional coping strategy and complex behaviour. The message is clear that pacing should enable increases in meaningful activities, as opposed to attempting to avoid symptoms. The survey findings have informed the development of an activity pacing framework to guide healthcare professionals on the multiple components of pacing. This will help to standardize and optimize treatments for chronic pain/fatigue and enable future investigations.

## 1 INTRODUCTION

A multidisciplinary, biopsychosocial approach is recommended to manage complex conditions of chronic pain/fatigue, including chronic low back pain, chronic widespread pain, fibromyalgia and chronic fatigue syndrome/myalgic encephalomyelitis (CFS/ME) (British Pain Society, 2013; Kamper et al., 2015; National Institute for Health and Care Excellence, 2007). Such conditions may be classified as somatic symptom disorders owing to the presence of physical symptoms (for example, pain and weakness) that disrupt daily functioning and are associated with altered thoughts, feelings or behaviours (American Psychiatric Association, 2013). In keeping with this classification, the impact of chronic pain/fatigue includes disability, anxiety, depression and altered behaviours such as fear avoidance, excessive persistence and overactivity–underactivity cycling (Abonie, Sandercock, Heesterbeek, & Hettinga, 2018; Kindermans et al., 2011; National Institute for Health and Care Excellence, 2007; van Koulil et al., 2010).

Patients may present with one or more somatic disorders due to overlapping symptoms (Davis, Kroenke, Monahan, Kean, & Stump, 2016; Tavel, 2015). Consequently, conditions of chronic pain/fatigue may be treated together, using holistic interventions that include physical and psychological therapies (American Psychiatric Association, 2013; Tavel, 2015). Specifically, rehabilitation programmes involve graded exercise, cognitive behavioural therapy (CBT), acceptance and commitment therapy (ACT) and mindfulness (British Pain Society, 2013; Goudsmit, Ho‐Yen, & Dancey, 2009; Larun, Brurberg, Odgaard‐Jensen, & Price, 2017; National Institute for Health and Care Excellence, 2007, 2016). Activity pacing is considered an important component of such strategies, and a core element of rehabilitation programmes (Beissner et al., 2009; Birkholtz, Aylwin, & Harman, 2004a; Booth et al., 2017; Nielson, Jensen, Karsdorp, & Vlaeyen, 2013; Torrance et al., 2011; Wallman, Morton, Goodman, Grove, & Guilfoyle, 2004).

Activity pacing aims to modify behaviours of avoidance, excessive persistence and overactivity–underactivity/boom–bust cycling. Boom‐bust cycling involves fluctuations between high activity levels (excessive persistence) which leads to increased symptoms and consequential days of low activity levels (Birkholtz et al., 2004a). Pacing encourages more consistent engagement in regular and meaningful activities, while reducing flare‐ups (Beissner et al., 2009; Birkholtz et al., 2004a; Nielson et al., 2013).

Pacing has been labelled using varying terminology—for example, activity modification, tailored pacing and activity scheduling. It has been described as adaptive pacing therapy (activities are undertaken within limited amounts of energy) (White et al., 2011) and the envelope theory (energy expenditure matches perceived energy levels) (Goudsmit & Howes, 2017). Such principles align with symptom contingency, in which activities are driven by perceived symptom levels, with the aim of avoiding symptoms/conserving energy (Racine, Jensen, Harth, Morley‐Forster, & Nielson, 2018). However, there have been minimal measurable improvements in function when pacing is described in these terms (Goudsmit et al., 2009; White et al., 2011). Alternatively, pacing has been described as Fordyce's operant approach (Fordyce, 1976). This quota‐contingent approach involves undertaking activities according to an amount/distance/goal with the aim of improving function (Nielson et al., 2013). The aim of pacing, to increase function or reduce the severity of symptoms, plays an important role in the efficacy of the strategy (Esteve et al., 2017; Hadzic, Sharpe, & Wood, 2017).

The individual facets of pacing may also influence whether patients benefit from it. Commonly cited facets of pacing include going slow and steady, breaking down tasks and using rest breaks (Cane, Nielson, McCarthy, & Mazmanian, 2013; McCracken & Samuel, 2007; White et al., 2007). As such facets may involve reductions in activities, it is perhaps unsurprising that pacing has been significantly associated with inactivity, avoidance and disability (Cuperus, Hoogeboom, Neijland, van den Ende, & Keijsers, 2012; Hadzic et al., 2017; McCracken & Samuel, 2007). Other facets of pacing include planning, consistency, setting goals and gradually increasing activities (Antcliff, Campbell, Woby, & Keeley, 2015; Birkholtz, Aylwin, & Harman, 2004b; Nielson et al., 2013). The effects of these facets have been less widely investigated.

To date, there is no consensus on the use and effects of different types of pacing. Adaptive pacing therapy for CFS/ME has been found to be ineffective (White et al., 2011); energy conservation for CFS/ME has improved fatigue, anxiety and self‐efficacy but not functional impairment (Goudsmit et al., 2009); pacing for chronic pain (breaking down tasks/using rest breaks) has been associated with improved psychological function, without improving disability or avoidance (Cane et al., 2013; McCracken & Samuel, 2007). Therefore, there is no widely used method of pacing that has consistently improved psychological and physical function among patients with chronic pain/fatigue.

Within the context of chronic pain/fatigue, there remains much debate into the types/uses of pacing, the aims, facets and clinical effects. In the absence of a strong evidence base or standardized framework, it follows that healthcare professionals, patients and researchers may interpret/implement pacing differently (Gill & Brown, 2009; Nielson et al., 2013). Here, we describe the first stage in the development of a comprehensive activity pacing framework which has wider clinical utility through its relevance for chronic pain and fatigue.

### 1.1 Aim

The aim of the present study was to explore current opinions and practices of pacing among multidisciplinary healthcare professionals in a nationwide survey. These findings informed the development of the initial activity pacing framework for healthcare professionals.

## 2 METHODS

### 2.1 Study design

An online national survey was designed to explore the wider opinions of multidisciplinary healthcare professionals on activity pacing.

### 2.2 Participants

Eligible participants included qualified healthcare professionals with a minimum of two years' postgraduate clinical experience of working in pain/fatigue services in National Health Service (NHS) trusts in England. Eligible healthcare professionals included physiotherapists, occupational therapists, psychological therapists, doctors and nurses.

### 2.3 Recruitment

A mapping exercise was undertaken, to identify NHS sites in England that provided multidisciplinary/biopsychosocial treatments for chronic pain/fatigue. The mapping exercise was developed from published audits of pain services, online NHS directories, CFS/ME regional networks and special interest groups. Local contacts were identified at each department and contacted to scope for interest.

The mapping exercise enabled purposive sampling to recruit a range of multidisciplinary healthcare professionals working across different sites, to gather a variety of opinions/experiences. Recruitment took place through targeted emails to local contacts and via a snowball effect as local contacts forwarded the survey to other healthcare professionals/special interest groups (Hayslett & Wildemuth, 2004). Local contacts were asked to report the number of healthcare professionals to whom the survey had been forwarded, to allow an estimation of recruitment rates.

### 2.4 Data collection

The research team (physiotherapist, health psychologist, quantitative/qualitative researchers and statistician) developed the online survey based on the aforementioned areas of confusion surrounding pacing that appear most frequently in the existing literature. The resulting survey contained 28 questions to capture the types/uses of pacing, aims, facets and perceived effects. The survey contained a combination of open‐ and closed‐ended questions. It was piloted on two healthcare professionals (a clinical psychologist and an occupational therapist), who advised on the content and format of the survey, and established that it took approximately 20 min to complete. The pilot data were not included in the final dataset. Participants were asked to complete the survey within 2 weeks, as per usual response times for online surveys (Hayslett & Wildemuth, 2004). Typical response rates to online surveys of 25–33% were envisaged, and a reminder was sent 2 weeks later to increase the response rate (Sánchez‐Fernández, Muñoz‐Leiva, & Montoro‐Ríos, 2012; Shih & Fan, 2009).

### 2.5 Data analysis

Quantitative data from the demographic and closed‐ended survey questions were analysed using IBM SPSS Statistics 24 (IBM SPSS Statistics Version 24: statistical software; IBM Corporation, Armonk, NY). Descriptive statistics summarized the representativeness of the participants and the frequencies of different aspects of pacing. Qualitative data from the open‐ended questions were organized using the NVivo11 program (QSR International Pty Ltd. Victoria, Australia) and analysed using thematic analysis (Braun & Clarke, 2006). Thematic analysis involves an iterative process of reading the information and coding themes (Braun & Clarke, 2006; Ritchie, Spencer, & O'Connor, 2003). It is considered to be a transparent method of qualitative analysis that can be repeated/retraced (Ritchie et al., 2003). It involves six stages: familiarization with the data, coding, searching for themes, reviewing themes, labelling/defining themes and writing up (Braun & Clarke, 2006). This analysis was performed by two members of the research team independently (D.A. and L.M.), and the results were discussed to reach a consensus for the themes and codes related to the open‐ended survey questions.

### 2.6 Ethical approval

Ethical approval was granted by the West of Scotland REC 5 (IRAS Ref: 16/WS/0209; Protocol version 1.0).

## 3 RESULTS

### 3.1 Participants

A total of 115 NHS trusts in England were identified as providing persistent pain services, and 53 provided CFS/ME services. Of these sites, 52 NHS trusts consented to receive the survey (excluding duplication of trusts). The survey was sent to 78 healthcare professionals as targeted emails, who forwarded the survey to at least 152 healthcare professionals. Therefore, a minimum of 230 healthcare professionals received the survey. The survey was open for 32 days.

Sixty‐five healthcare professionals responded to the initial survey and 32 responded to the reminder, resulting in 97 respondents (estimated total response rate = 42.2%). Reasons for exclusion were: two participants had <2 years' clinical experience in chronic pain/fatigue, two participants were based in Northern Ireland and one was based in private practice. Subsequently, data from 92 respondents across 48 trusts were analysed.

### 3.2 Demographics

Of the healthcare professions, physiotherapists formed the largest group (*n* = 45, 49.5%); the majority of participants were female (*n* = 70, 77.8%); a third of participants had 10–14 years' experience of working in chronic pain/fatigue (*n* = 29, 32.2%); and outpatient departments were the most frequent clinical setting (*n* = 50, 36.8%) (see Table 1).

**Table 1:** Participants' demographics

Demographics		Number (%)
Gender^a^	Male	20 (22.2)
	Female	70 (77.8)
Age^a^	20–29 years	2 (2.2)
	30–39 years	25 (27.2)
	40–49 years	34 (37.0)
	50–59 years	25 (27.2)
	60+ years	6 (6.5)
Healthcare profession^a^	Nurse	4 (4.4)
	Doctor	4 (4.4)
	Physiotherapist	45 (49.5)
	Occupational therapist	30 (33.0)
	Clinical psychologist	7 (7.7)
	Other: Cognitive behavioural therapist	1 (1.1)
Postgraduate experience in chronic pain/fatigue^a^	2–4 years	12 (13.3)
	5–9 years	20 (22.2)
	10–14 years	29 (32.2)
	15–19 years	17 (18.9)
	20–24 years	6 (6.7)
	25–29 years	4 (4.4)
	30+ years	2 (2.2)
Clinical base^a^, ^b^	Outpatients	50 (36.8)
	Inpatients	10 (7.4)
	Community	21 (15.4)
	Primary care	6 (4.4)
	Secondary care	28 (20.6)
	Tertiary care	18 (13.2)
	Other	3 (2.2)

a Participants could choose not to answer any of the demographic questions.

b Participants could select more than one answer.

### 3.3 Quantitative findings

#### 3.3.1 Uses/types of pacing

Pacing was highly utilized by healthcare professionals: 83 participants (90.2%) taught pacing as a self‐management strategy and 61 participants (66.3%) taught pacing in rehabilitation programmes. It was instructed as part of graded exercise/activity by 74 participants (80.4%) and CBT by 45 participants (48.9%). Fifty‐one participants (55.4%) used pacing alongside mindfulness, and 37 participants (40.2%) incorporated pacing into ACT. Thirteen participants (14.1%) believed that pacing was helpful for all patients, and 64 (69.6%) that it was helpful for the majority of patients. Three participants (3.3%) believed that pacing was only helpful for a minority of patients.

**Figure 1:**
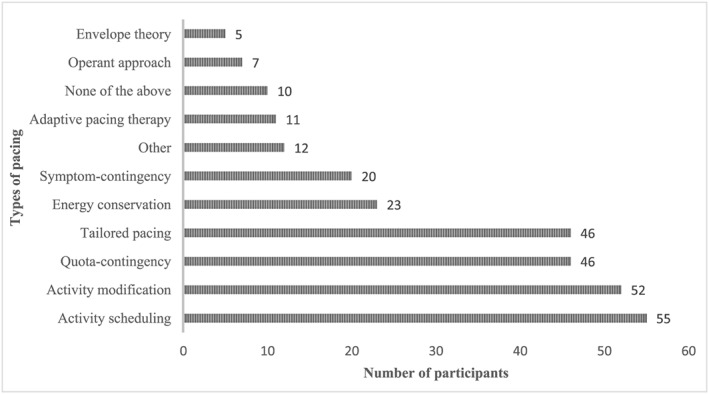
Bar chart of the types of pacing. Participants could select more than one answer

The most frequently used types of pacing were activity scheduling (*n* = 55, 59.8%), activity modification (*n* = 52, 56.6%), quota‐contingent pacing (*n* = 46, 50.0%) and tailored pacing (*n* = 46, 50.0%). The least endorsed types of pacing were the envelope theory (*n* = 5, 5.4%), operant approach (*n* = 7, 7.6%) and adaptive pacing therapy (*n* = 11, 12.0%). Participants could provide more than one answer; however, a number of participants were unfamiliar with some terminology. Few participants commented that they used term “activity management” owing to connotations of avoidance with “pacing”. Participants' responses are summarized in Figure 1.

#### 3.3.2 Aims of pacing

Of the aims of pacing, the three most highly ranked priorities were for the achievement of meaningful activities (score = 220, 24.5% of votes), to increase self‐efficacy (score = 128, 14.2%) and to manage symptoms (score = 121, 13.5%). The three least supported aims of pacing were to conserve energy (score = 1, 0.1%), to reduce symptoms (score = 15, 1.7%) and to improve mood (score = 20, 2.2%). Participants suggested other aims of pacing, including to improve satisfaction with activities, challenge beliefs about activities and improve sense of control/choice (see Table 2).

**Table 2:** Ranked scores of the aims of pacing

Pacing aim	Number of participants selecting each priority rating^a^				Score for each pacing aim (% of all rankings)
	4 = most important	3	2	1 = least important	
1. Achievement of meaningful activities	39	12	10	9	220 (24.5)
2. Increase self‐efficacy	12	15	11	13	128 (14.2)
3. Manage symptoms	18	6	10	11	121 (13.5)
4. Change activity behaviours	6	10	7	8	76 (8.5)
5. Reduce fear avoidance	2	10	14	8	74 (8.2)
6. Reduce disability	3	11	9	10	73 (8.1)
7. Regulate activity levels	2	8	7	7	53 (5.9)
8. Increase activity levels	2	6	9	5	49 (5.5)
9. Prevent a flare‐up	2	4	5	5	35 (3.9)
10. Acceptance of symptoms	2	4	6	2	34 (3.8)
11. Improve mood	0	2	3	8	20 (2.2)
12. Reduce symptoms	2	2	0	1	15 (1.7)
13. Conserve energy	0	0	0	1	1 (0.1)

a Participants were asked to select their top four ranked answers.

#### 3.3.3 Facets of pacing

The most highly endorsed facets of pacing were “breaking down tasks” (*n* = 91, 98.9%), “spreading out activities over time” (*n* = 89, 96.7%), “switching between different types of activities”, “allowing flexibility with activities” and “undertaking some level of activities despite symptoms” (all *n* = 87, 94.6%). The least endorsed facets of pacing were ‘stopping activities when symptoms increase' (*n* = 6, 6.5%), “avoidance of activities that aggravate symptoms” (*n* = 11, 12.0%) and “working below tolerance levels” (*n* = 31, 33.7%). Participants suggested additional facets of balancing activities, addressing self‐imposed rules, having psychological and behavioural flexibility, and choosing when to stop/change activity (see Table 3).

**Table 3:** Participants' votes of endorsement on the different facets of pacing

	Facets of pacing^a^	Number of participants (% of participants)
1	Breaking down tasks	91 (98.9)
2	Spreading out activities over time	89 (96.7)
3	Switching between different types of activity	87 (94.6)
4	Allowing flexibility with activities	87 (94.6)
5	Undertaking some level of activities despite symptoms	87 (94.6)
6	Planning activities in advance	86 (93.5)
7	Delegating tasks	85 (92.4)
8	Setting realistic goals	85 (92.4)
9	Learning from experience	85 (92.4)
10	Not doing too much on a “good” day	83 (90.2)
11	Having scheduled breaks during activities	82 (89.1)
12	Being able to say “no”	82 (89.1)
13	Undertaking meaningful activities	82 (89.1)
14	Doing some activity on a “bad” day	82 (89.1)
15	Finding a baseline of activities	82 (89.1)
16	Acceptance of abilities	81 (88.0)
17	Setting meaningful goals	81 (88.0)
18	Prioritizing activities	81 (88.0)
19	Asking for help	80 (87.0)
20	Gradually increasing activities	80 (87.0)
21	Alternating between activity and rest	79 (85.9)
22	Changing positions	79 (85.9)
23	Having consistent levels of activities	71 (77.2)
24	Setting quotas (time/amounts) of activities	69 (75.0)
25	Persistence with activities/modified activities	69 (75.0)
26	Having a routine	65 (70.7)
27	Using relaxation	64 (69.6)
28	Using mindfulness	64 (69.6)
29	Stopping activities before symptoms increase	59 (64.1)
30	Going slow and steady	51 (55.4)
31	Spending less time on activities in order to do them more frequently	49 (53.3)
32	Working within a perceived percentage of energy	38 (41.3)
33	Working below tolerance levels	31 (33.7)
34	Avoidance of activities that aggravate symptoms	11 (12.0)
35	Stopping activities when symptoms increase	6 (6.5)

a Participants could vote on all facets.

### 3.4 Qualitative findings

#### 3.4.1 Effects of pacing

Three themes and 12 subthemes emerged from the thematic analysis of the open‐ended question regarding participants' views on the effects of pacing. The three themes were: benefits of pacing, disadvantages of pacing, and pacing approaches/complementary therapies.

“Benefits of pacing” included short‐term benefits, such as establishing baselines, improved symptom management and psychological well‐being. Longer‐term benefits included developing routines and setting goals to incorporate meaningful/desirable activities to improve quality of life. Pacing encouraged a modification of self‐expectations/beliefs about activities, a reduction in fear of symptoms/activities, less boom–bust cycling and improved cumulative activity levels. Pacing could enable a sense of choice and control over activities and allow for planned events due to more predictable symptoms (see Table 4).

**Table 4:** Thematic analysis of healthcare professionals' views on the effects of pacing on patients

Theme	Subthemes	Codes and examples
Benefits of pacing	Short‐term effects	**Prevent over‐exertion:** *“giving very active people ‘permission' to break up activities”* [physiotherapist] **Challenges avoidance:** *“challenging held beliefs about whether or not an activity is possible”* [physiotherapist] **Find baselines:** *“establish baseline tolerance”* [clinical psychologist]; *“sustainable activity levels”* [occupational therapist] **Self‐management:** *“actively manage their pain/energy levels and not just be ‘victims'”* [occupational therapist] **Self‐learning:** *“better understanding of previous patterns of activity”* [occupational therapist]
	Long‐term effects	**Helpful in the long term:** *“gives individuals choice as to how much to do: less or more”* [physiotherapist] **Improved prioritization:** *“prioritize tasks which can help to improve quality of life”* [physiotherapist] **Increase meaningful activity:** *“meaningful engagement in valued activities”* [physiotherapist] **Improve quality of life (QoL):** *“focus on what activities have true value for improved QoL”* [physiotherapist] **Provide guidance/structure:** *“framework for gradually increasing activity over time”* [physiotherapist] **Reduce boom–bust:** *“more consistent levels of activity often result in better QoL”* [physiotherapist] **Setting goals:** *“set new goals and challenges for the future”* [physiotherapist]
	Effects on activities	**Increase activity:** *“increase and sustain more consistent activity levels”* [physiotherapist] **Balance activities:** *“schedule a more balanced range of activities”* [occupational therapist] **Reduce boom–bust:** *“avoid overactivity/underactivity cycling”* [physiotherapist] **Improve endurance:** *“stamina and endurance to activity without excessive and disruptive pain flares”* [physiotherapist]
	Effects on symptoms	**Manage symptoms:** *“lessen patients' pain intensity”* [physiotherapist]; *“improved energy levels”* [occupational therapist] **Reduce flare‐ups:** *“reduce intensity and frequency of flare ups*” [physiotherapist]*; “stabilization of symptoms”* [occupational therapist] **Reduce medication:** *“behavioural form of pain control”* [physiotherapist]
	Effects on mood	**Improve mood:** *“self‐care and self‐compassion”* [occupational therapist]; *“fosters a sense of hope”* [physiotherapist] **Acceptance:** *“less of a battle with their pain”* [nurse]; *“improved satisfaction with activity”* [occupational therapist] **Improve frustration:** *“reduces overactivity/underactivity‐related frustration and demoralisation”* [clinical psychologist] **Greater sense of control:** *“feel more in control and … more able to make decisions about life”* [clinical psychologist] **Improve confidence:** *“promote self‐efficacy”* [occupational therapist]; *“confidence to cope with pain”* [doctor]
Disadvantages of pacing	Short‐term effects	**Reduce activity:** *“patients can feel that they are achieving less”* [physiotherapist] **Avoidance:** *“some say they are pacing, when in fact they are just decreasing or avoiding activities”* [physiotherapist] **Frustration:** *“heighten a sense of a patient being restricted by their pain”* [clinical psychologist]; *“punitive regime”* [physiotherapist]
	Long‐term effects	**Prevent progression:** *“scared to move forward with graded exercise as it takes them out of their comfort zone”* [occupational therapist] **Reduce activity:** *“doing less than preferred”* [physiotherapist]
	Effects on activities	**Prevent progression:** *“stagnation if too rigid”* [physiotherapist]; *“ceiling effect”* [physiotherapist] **Reduce activity:** *“pacing is misrepresented or misunderstood as ‘listening to your body'”* [clinical psychologist]
	Effects on symptoms	**Symptom‐contingency:** *“if it is used to conserve energy … can reduce occupational performance”* [occupational therapist] **Worsen symptoms:** *“it is also important that people are not given the message that activity is dangerous, or that they must pace themselves to prevent their condition worsening, as this can lead to unhelpful anxieties* [clinical psychologist]
	Effects on mood	**Avoidance:** *“maladaptive pacing is a form of emotional avoidance”* [physiotherapist] **Frustration:** *“don't like leaving things unfinished”* [occupational therapist]; *“another frustrating control strategy*” [physiotherapist];*“disruptive”* [physiotherapist] **Worsen mood:** *“worries about getting it* [pacing] *right”* [occupational therapist]; *“stigmatized”* [physiotherapist]; *“depressing”* [occupational therapist]
Pacing approaches and complementary therapies	Type of pacing	**Type of pacing:** *“traditional pacing is not helpful”* [physiotherapist]; *“adaptive pacing … people getting stuck at their current level … incremental pacing … aims for gradual increase”* [occupational therapist]; *“mindful approach”* [physiotherapist]
	Flexibility	**Flexibility:** *“people who pace well can be flexible and choose to ‘overdo' things without fear”* [physiotherapist] **Tailored pacing:** *“not a ‘one size fits all' strategy”* [occupational therapist]; *“tailoring to each patient's needs”* [occupational therapist]

“Disadvantages of pacing” involved the possible struggle for patients to adapt/limit activities in the short term to find baselines. Limiting activities may also be misinterpreted as avoidance. Conversely, there may be initial increases in symptoms for patients using pacing to re‐engage in activities. Potential longer‐term disadvantages of pacing included preventing progression among patients who felt safe at their baselines. Pacing may have negative effects on mood, such as being restrictive or depressing, especially if pacing aimed to reduce pain/save energy.

“Pacing approaches/complementary therapies” involved participants' opinions that the type of pacing determined whether it would be beneficial or harmful. Perceptions of “traditional pacing” or adaptive pacing were suggested as being potentially harmful/preventing progress. Many participants believed that pacing needed to be tailored and flexible to be beneficial.

#### 3.4.2 Existing pacing guides

There was no pacing guide that was consistently used among participants. They reported limitations of existing pacing information as it appeared to be based on anecdotal findings, and different resources yielded conflicting messages. Participants reported advantages and disadvantages both of condition‐specific and generic guides, including patient preference and usability. Across pain and fatigue services alike, participants commented that they instructed pacing similarly, as a quota‐contingent strategy that addressed avoidance behaviours and encouraged graded activity.

#### 3.4.3 Developing a new activity pacing framework

Participants believed that developing a comprehensive pacing framework would help to standardize pacing for chronic pain/fatigue. The new framework should not be prescriptive or formulaic; instead, patients should select the facets of pacing that are useful for them. Participants encouraged choice regarding when to pace and when overexertion was necessary or worthwhile. A new pacing framework needed to define the type of pacing and reflect the complex nature of pacing.

## 4 DISCUSSION

To our knowledge, this is the first national and multidisciplinary survey into activity pacing that has been undertaken with the specific purpose of developing an activity pacing framework for chronic pain/fatigue. The survey showed that activity pacing continues to be frequently instructed by healthcare professionals. The response to the survey was demonstrative of the continued interest and discussion surrounding the different uses/types, aims, facets and effects of pacing.

### 4.1 Uses/types of pacing

Participants used differing terminology relating to pacing, which may illustrate some variances in its delivery. However, more participants advocated quota‐contingent than symptom‐contingency pacing. Similarly, other literature advocates quota‐contingency/operant approaches to enable rehabilitative interventions that increase individuals' sense of control over activities, rather than being controlled by symptoms as per symptom‐contingency/energy conservation (Birkholtz et al., 2004b; Fordyce, 1976; Nielson et al., 2013; Racine et al., 2018).

Pacing was considered to complement other coping strategies, such as graded exercise/activity. Few participants commented that pacing was disparate to graded activity/”pacing up”. There is ongoing debate as to whether pacing involves graded activity, and this may depend on the pacing method (Nielson et al., 2013). There are also discussions around whether increases in activity are a *facet* or a *goal* of pacing (Andrews & Deen, 2016). The findings from our survey suggest that gradual increases in activity have the potential to be both.

Many participants considered pacing as a key component of CBT, as per earlier recommendations of behavioural therapy for chronic pain (Fordyce, 1976). Accordingly, they suggested that pacing could help patients to change unhelpful behaviours/self‐inflicted rules. They believed that pacing could be congruent with mindfulness and ACT, provided that it did not aim to control symptoms. Likewise, Scott‐Dempster, Toye, Truman, and Barker (2014) found that pacing encouraged a sense of choice, and suggested compatibility between pacing and ACT. Similar to ACT and mindfulness, participants in this survey commented that pacing involved flexibility, promotion of meaningful activities, acceptance and active decision‐making (McCracken, Sato, & Taylor, 2013).

### 4.2 Aims of pacing

The most endorsed aim of pacing was for the achievement of meaningful activities, in agreement with a previous survey (Cuperus et al., 2016). The second most endorsed aim of pacing was to increase self‐efficacy. The importance of self‐efficacy as a correlate and mediator in chronic pain is established (Woby, Urmston, & Watson, 2007), and pacing has previously been considered to improve self‐efficacy (Scott‐Dempster et al., 2014). The least supported aims of pacing were to conserve energy/reduce symptoms. Such aims are similar to the energy envelope theory/energy conservation and adaptive pacing therapy (Racine et al., 2018; White et al., 2007). When pacing has the aim of reducing symptoms, avoidant behaviours may present (Esteve et al., 2017). Therefore, it is crucial to clarify the aims of pacing within the activity pacing framework.

### 4.3 Facets of pacing

The three most favoured facets of pacing included the widely cited and traditional components of breaking down tasks, spreading out activities and switching between activities (Andrews, Strong, Meredith, Gordon, & Bagraith, 2015; Birkholtz et al., 2004a). Notably, the fourth most endorsed facet of pacing was being flexible with activities, which corresponds with other literature (Scott‐Dempster et al., 2014). The authors suggest that the emergence of flexibility as a component of pacing may reflect participants' recognition of unsuccessful pacing regimes that have historically been too prescriptive, together with the growing use of ACT and mindfulness.

The least endorsed facets included themes of stopping/avoiding activities according to symptoms and working below tolerance levels. Such facets align with adaptive pacing therapy and energy conservation (Racine et al., 2018; White et al., 2007). These facets may potentially lead to a reduction/avoidance of activities, and may be ineffective for improving the management of long‐term conditions (White et al., 2011). The activity pacing framework will clarify the facets of pacing that are recommended as a rehabilitative coping strategy.

### 4.4 Effects of pacing

Most participants perceived the benefits of pacing to include achieving more meaningful activities/a wider variety of activities in the long term. However, pacing was considered to pose challenges in the short term if it involved limiting/adapting activities while finding baselines. Caution was required to ensure that pacing did not result in longer‐term reductions in the number of activities or in stagnation. Such negative effects of pacing were considered more likely if the aims were to prevent symptoms/conserve energy. Similarly, Goudsmit et al. (2009) failed to find significant improvements in function or depression when pacing involved the limitation of activities/activity progression.

### 4.5 Lack of guidance

Despite the high utility of pacing, there was an absence of an agreed and evidence‐based pacing guide. The development of a framework that is applicable to both chronic pain and fatigue was welcomed more than rebuked among participants. This may reflect the frequent clinical presentation and known coexistence of conditions of chronic pain/fatigue (Tavel, 2015).

### 4.6 Strengths and limitations of the study

There was an exceptional response to this national survey from multidisciplinary healthcare professionals in terms of the number of participants and detailed responses. Unfortunately, due to the method of recruitment, true response rates could be calculated. There were fewer trusts delivering services for CFS/ME than for pain. However, similar views towards rehabilitative approaches of pacing emerged across pain and fatigue disciplines from participants' responses. Quantitative and qualitative data analyses of participants' responses took place, with the researchers blinded to participants' demographic information to reduce researcher bias. Given the uneven proportion of different healthcare professionals, it is important that future work further explores how opinions might differ across a broader group of healthcare professionals.

The initial recruitment of participants was limited to those English NHS trusts with identifiable websites, and the dissemination of the survey depended on gate keepers who received the initial contact. Implementing a snowball recruitment method aimed to encourage wider participation.

## 5 CONCLUSION

Activity pacing continues to be frequently utilized as a coping strategy for chronic pain/fatigue. However, there is currently no standardized framework to guide healthcare professionals. Pacing is considered to be beneficial for patients, but the benefits appear to be dependent on the interpretation of pacing. For pacing to be rehabilitative, the aims of pacing are suggested to include achieving meaningful activities, rather than avoiding symptoms. In this survey, concepts of flexibility and choice were paramount, which contrasts some traditional interpretations of pacing.

Informed by the findings of this survey and existing literature, we have developed the first draft of an activity pacing framework for multidisciplinary healthcare professionals. The next phase of work involves engaging stakeholders (patients and healthcare professionals) in a consensus method to refine the framework. The development of an activity pacing framework will help to provide an up‐to‐date, multidimensional and evidence‐based framework that may clarify the concept of activity pacing and facilitate future investigations into the effects of this complex strategy.

## CONFLICTS OF INTEREST

The authors have no conflicts of interest to declare.

## FUNDING INFORMATION

This study is funded by Health Education England/National Institute for Health Research (HEE/NIHR) [Clinical Lectureship (ICA‐CL‐2015‐01‐019)]. The views expressed are those of the author(s) and not necessarily those of the NIHR or the Department of Health and Social Care.
